# Usefulness of Automatic Speech Recognition Assessment of Children With Speech Sound Disorders: Validation Study

**DOI:** 10.2196/60520

**Published:** 2025-01-14

**Authors:** Do Hyung Kim, Joo Won Jeong, Dayoung Kang, Taekyung Ahn, Yeonjung Hong, Younggon Im, Jaewon Kim, Min Jung Kim, Dae-Hyun Jang

**Affiliations:** 1 Department of Rehabilitation Medicine Incheon St Mary’s Hospital, College of Medicine The Catholic University of Korea Seoul Republic of Korea; 2 Department of English Language and Literature Korea University Seoul Republic of Korea; 3 MediaZen Seongnam-si, Gyeonggi-do Republic of Korea; 4 Department of Special Education Dankook University Youngin-si, Gyeonggi-do Republic of Korea

**Keywords:** speech sound disorder, speech recognition software, speech articulation tests, speech-language pathology, child

## Abstract

**Background:**

Speech sound disorders (SSDs) are common communication challenges in children, typically assessed by speech-language pathologists (SLPs) using standardized tools. However, traditional evaluation methods are time-intensive and prone to variability, raising concerns about reliability.

**Objective:**

This study aimed to compare the evaluation outcomes of SLPs and an automatic speech recognition (ASR) model using two standardized SSD assessments in South Korea, evaluating the ASR model’s performance.

**Methods:**

A fine-tuned wav2vec 2.0 XLS-R model, pretrained on 436,000 hours of adult voice data spanning 128 languages, was used. The model was further trained on 93.6 minutes of children’s voices with articulation errors to improve error detection. Participants included children referred to the Department of Rehabilitation Medicine at a general hospital in Incheon, South Korea, from August 19, 2022, to June 14, 2023. Two standardized assessments—the Assessment of Phonology and Articulation for Children (APAC) and the Urimal Test of Articulation and Phonology (U-TAP)—were used, with ASR transcriptions compared to SLP transcriptions.

**Results:**

This study included 30 children aged 3-7 years who were suspected of having SSDs. The phoneme error rates for the APAC and U-TAP were 8.42% (457/5430) and 8.91% (402/4514), respectively, indicating discrepancies between the ASR model and SLP transcriptions across all phonemes. Consonant error rates were 10.58% (327/3090) and 11.86% (331/2790) for the APAC and U-TAP, respectively. On average, there were 2.60 (SD 1.54) and 3.07 (SD 1.39) discrepancies per child for correctly produced phonemes, and 7.87 (SD 3.66) and 7.57 (SD 4.85) discrepancies per child for incorrectly produced phonemes, based on the APAC and U-TAP, respectively. The correlation between SLPs and the ASR model in terms of the percentage of consonants correct was excellent, with an intraclass correlation coefficient of 0.984 (95% CI 0.953-0.994) and 0.978 (95% CI 0.941-0.990) for the APAC and UTAP, respectively. The *z* scores between SLPs and ASR showed more pronounced differences with the APAC than the U-TAP, with 8 individuals showing discrepancies in the APAC compared to 2 in the U-TAP.

**Conclusions:**

The results demonstrate the potential of the ASR model in assessing children with SSDs. However, its performance varied based on phoneme or word characteristics, highlighting areas for refinement. Future research should include more diverse speech samples, clinical settings, and speech data to strengthen the model’s refinement and ensure broader clinical applicability.

## Introduction

### Background

Speech sound disorders (SSDs) are common communication disorders among children, wherein they experience difficulty producing speech sounds or using them correctly when compared to children of the same age [1]. In South Korea, approximately 44.1% of children undergoing speech therapy have SSDs [2], and around 9% of 6-year-old children exhibit articulation problems [3].

SSDs among children are evaluated by prompting spontaneous speech using single-word naming tasks, sentence repetition tasks, and connected speech tasks [4]. In South Korea, standardized assessment tools such as the Assessment of Phonology and Articulation for Children (APAC) and Urimal Test of Articulation and Phonology (U-TAP) have been predominantly used to assess children with SSDs in the clinical setting [5,6]. The percentage of correct consonants (PCC, %) serves as the primary criterion for diagnosing SSDs. Aside from PCC, speech error patterns must also be examined and analyzed throughout the entire transcription to guide the direction of intervention for children.

Nonetheless, traditional evaluation methods face two primary issues. First, these methods are time-consuming [4,7]. In fact, the process by which speech-language pathologists (SLPs) evaluate children with SSDs lasts approximately 2-2.5 hours, due to the need for accurate transcription and analysis of individual speech error patterns [7]. The second issue involves reliability, which has been attributed to slight differences in results depending on the tester, potentially arising from variations in the tester’s skill level and the test environment [8,9].

Consensus among the SLPs is influenced by patterns of speech errors, with certain patterns being more readily recognizable than others. Studies have shown that misarticulations involving only one feature change are usually ignored, whereas those with two or more feature changes are readily recognized [9,10]. Moreover, evidence suggests that identifying misarticulations of vowels is more challenging than that of consonants [11], with combinations of both further increasing the difficulty [12]. Regarding consonants, listeners can more consistently detect voicing errors than they would changes in the place of articulation [13]. In particular, Klein et al [14] showed diversity in the ratings of /r/ production among SLPs, due to difficulties in auditory-perceptual discrimination. These studies suggest that SLPs find it more challenging to transcribe incorrectly produced phonemes than those produced correctly. Thus, streamlined and standardized evaluations are essential for achieving accurate and consistent assessments of speech sound errors in children with SSDs.

### Prior Studies

Automating the process of transcribing children’s speech and analyzing patterns of speech errors could enhance the efficiency and reliability of the evaluation process benefiting both patients and SLPs [8]. Investigations on automatic speech recognition (ASR) for assessing children with SSDs have used various approaches to enhance accuracy and reliability [15-18].

Traditional ASR models using the hidden Markov model (HMM) are divided into three parts: Lexicon, Acoustic, and Language models. Each part is modeled independently from each other. Suanpirintr and Thubthong [16] obtained a phoneme error rate (PER; the number of incorrectly recognized phonemes divided by the total number of phonemes) of 51.5% for dysarthria in Thailand using an HMM. Mazenan et al [17] also used an HMM for Malay SSDs, reporting low PER rates (approximately 3%) on various isolated phonemes; however, their results were limited to Malay alveolar sounds. Lee et al [18] evaluated articulation using an HMM targeting patients with dysarthria in South Korea and confirmed that their PCC results were strongly correlated with those of SLPs; however, no information was provided regarding the agreement between individual phonemes in the actual transcription results. Even though HMM has been used in many studies, developing HMM-based models requires considerable time and expertise, involving detailed data analysis and careful model validation.

Building on the strengths and limitations of HMM-based models, recent advancements have led to more advanced approaches like end-to-end models. These models simplify the process by directly linking inputs to outputs, removing the need for separate steps. As a preliminary investigation to this study, Ahn et al [19] confirmed that the end-to-end model (Wav2Vec2-XLS-R-1B) is suitable for evaluating children with SSDs even with limited training datasets for detecting speech errors. Owing to the pretrained framework of the Wav2Vec2-XLS-R-1B, it effectively learns from a wide variety of waveform contexts [19]. This pretraining allows the model to generalize well from smaller datasets and enables the model to better recognize and more accurately process Korean phonemes. They achieved approximately 90% accuracy in recognizing speech from children with SSDs in 73 Korean words using only about 1.5 hours of training data and without a language model. This suggests that sufficient accuracy can be attained in speech recognition models with low-resource languages to identify speech errors by using an end-to-end model.

### Objective

This study aimed to (1) compare the evaluation results of the SLPs and the ASR model using South Korea’s standardized SSD evaluation tools, specifically the APAC and U-TAP, and (2) analyze the cases of recognition errors occurring frequently in the ASR model.

## Methods

### Ethical Considerations

This study was conducted in accordance with the Declaration of Helsinki and was approved by the Institutional Review Board of the Catholic University of Korea, Incheon St. Mary’s Hospital (protocol code OC22OISI0041; received approval on August 19, 2022). All participants provided informed consent and were compensated with KRW 50,000 (approximately US $40) for their participation. Written informed consent was obtained from all parents. For children aged 7 years and older, additional written assent was collected. Research data were securely stored on hospital servers, separate from personally identifiable information except for study identification numbers. Access to and analysis of data were limited exclusively to preapproved researchers. This paper and any supplementary materials contain no identifiable images or personal information regarding participants.

### Recruitment

Participants were recruited between August 19, 2022, and June 14, 2023, from children referred for speech and language evaluations in the Department of Rehabilitation Medicine at Incheon St. Mary’s Hospital. Eligible participants were native Korean-speaking children younger than 18 years of age who were referred primarily due to concerns with articulation. Exclusion criteria included (1) intellectual disabilities or autism spectrum disorders that impaired effective engagement in the assessment; and (2) motor speech disorders, fluency disorders, a history of cleft palate, or severely reduced speech intelligibility that would hinder accurate transcription. Only children meeting these criteria, and whose parents provided consent, were included in the study.

We included 30 children with suspected SSD (20 male and 10 female children; aged 3-7 years). The age and sex distribution of the participants were as follows: aged 3 years (2 male children), aged 4 years (10 male and 5 female children), aged 5 years (4 male and 1 female children), aged 6 years (2 male and 3 female children), and aged 7 years (2 male and 1 female children). The sample size was determined based on the median value observed in previous studies [20]. Additional participant information is provided in Multimedia Appendix 1.

### ASR Model

The ASR model used in this study is an end-to-end model (Wav2Vec2-XLS-R-1B) originally provided by Facebook (Meta) as an open-source model [21] and modified by Ahn et al [19] to enhance its ability to detect speech error well in Korean children.

It was originally developed through training with 436,000 hours of data from adult voice databases, encompassing 128 languages. To enhance the model’s ability to detect articulation errors, Ahn et al [19] used 93.6 minutes of speech data comprising 73 words from the APAC and U-TAP, and an additional 12 words, yielding a total of 6935 words from 95 children. The additional 12 words were selected during the engine fine-tuning stage to include phonemes and contexts not originally covered in the APAC or U-TAP. Detailed information regarding the word list for the model training can be found in Multimedia Appendix 2 [5,6].

### Evaluation of Model Performance

This study used two Korean standardized SSD tests, namely the APAC and U-TAP, which comprised 37 and 30 words, respectively, with 6 words overlapping between the two tests. The examinations and recordings were conducted in the speech therapy room at Incheon St. Mary’s Hospital by three experienced SLPs (DHK, JWJ, and DK), all of whom were trained in the assessment and treatment of children with SSDs. Although the facility was not completely soundproof, it was conducted in an environment where external noise was controlled.

During the test, the participants were asked to name pictures, and their responses were recorded using an iPhone X (Apple). In the recording process, a sufficient pause of 500-1000 ms was ensured between each target word. The recordings were saved in WAV format and converted to a 16 kHz sampling rate to verify the recognition results. Each target word was attempted at least twice, allowing for the exclusion of words with quality issues, such as noise or insufficient volume. After recording, files were reviewed, and the most suitable file was selected for analysis.

**Figure 1:**
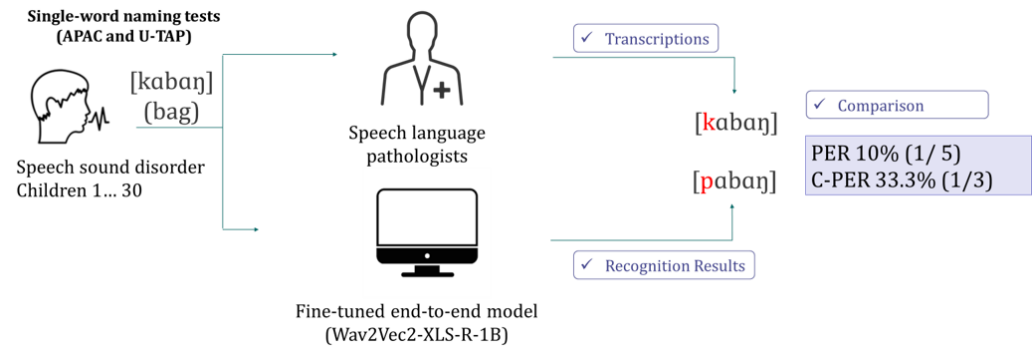
Comparison of the transcription results between the ASR model and the SLPs. APAC: Assessment of Phonology and Articulation for Children; ASR: automatic speech recognition; C-PER: consonant phoneme error rate; PER: phoneme error rate; SLP: speech-language pathologist; U-TAP: Urimal Test of Articulation and Phonology.

To ensure transcription accuracy, each word spoken by the participants in Korean was independently transcribed by two SLPs, and any disagreements were resolved by a third party for final decision-making. Transcription agreement between the two SLPs across all phonemes was 95% and 93.80% for the APAC and U-TAP, respectively, based on a 10% random sample of the data. The transcriptions created by the model were then compared to those obtained by the SLPs (Figure 1).

### Variables

#### PER

The PER (%) has been used to determine the difference in the overall phoneme when comparing the transcription results of the SLPs and ASR model. The consonant PER (C-PER, %) was examined separately to analyze consonant errors in the ASR model.



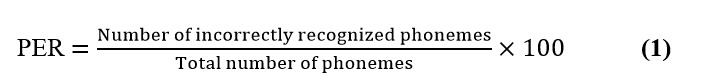





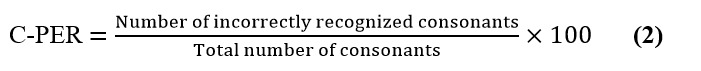



PER and C-PER between the SLPs and the ASR model were conducted across all phonemes, regardless of whether they are target phonemes. This comprehensive approach is crucial given that the accuracy of the entire transcription is essential for assessing the clinical utility of the ASR.

#### Common ASR Disagreements

This count encompasses discrepancies between the SLPs and the ASR model, including instances where the SLPs flagged correct articulations that were identified as misarticulations by the ASR model, as well as cases where the SLPs identified misarticulations within the set that the ASR model failed to match. This indicator assesses which test words (or phonemes) differed considerably in evaluation between the SLPs and the ASR model during tests.

#### Target PCC

The PCC (%) is a metric used to assess consonant articulation accuracy. In this study, “target PCC” specifically refers to the percentage of correct consonant sounds produced for the target phonemes, in each word test (ie, the APAC and U-TAP). Additional information about the target phonemes, and the number of production opportunities are provided in Multimedia Appendix 3.



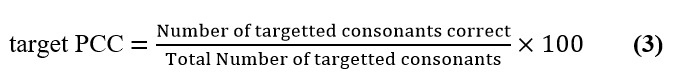



#### Severity

The *z* scores discussed in this study were based on the target PCC, which is used to evaluate the severity of SSDs. These *z* scores were calculated from the children’s assessments conducted by both the ASR model and the SLPs, after which the severity was compared between the two.

### Statistical Analysis

All data collected in this study were analyzed using SPSS (version 25.0; IBM Corp) for Windows. Descriptive statistics were used to describe the general characteristics of the evaluation results. The interrater reliability was calculated using the intraclass correlation coefficient (ICC) with a two-way random model, which served as the reliability coefficient for assessing the agreement on target PCC between the SLPs and the ASR model, using the absolute agreement method. Statistical significance was set at .05. The interpretation of the ICC was based on Landis and Koch [22].

## Results

### PERs

The PER, which indicated that the percentage of phonemes where the transcriptions by the SLPs and the ASR model differed among total phonemes, were 8.42% (457/5430) and 8.91% (402/4514) for the APAC and U-TAP, respectively. Additionally, the C-PER was 10.58% (327/3090) and 11.86% (331/2790) for APAC and U-TAP, respectively. The APAC uses a total of 174 phonemes, whereas the U-TAP consists of a total of 151 phonemes.

### Common ASR Disagreements

#### Disagreements in Correct Articulations

An average of 2.60 (SD 1.54) and 3.07 (SD 1.39) phonemes per child were transcribed as correctly produced by the SLPs but incorrectly produced by the ASR model, totaling 78 and 92 instances among 30 children, in the APAC and U-TAP, respectively. Table 1 presents the percentage of phonemes that were judged by the SLPs as correctly produced but identified as incorrectly produced by the ASR model. Table 2 lists the types of disagreements that occurred three or more times.

**Table 1:** The number of disagreements between the ASR^a^ and the SLPs^b^ for phonemes that were judged by the SLPs as correctly produced.

		Plosives, n/N^c^ (%)	Nasals, n/N (%)	Fluids, n/N (%)	Fricatives, n/N (%)	Affricatives, n/N (%)
**APAC^d^**						
	Syllable-initial word-initial	11/381 (2.89)	0/115 (0)	—^e^	2/143 (1.40)	4/84 (4.76)
	Syllable-initial word-medial	5/407 (1.23)	2/83 (2.41)	2/27 (7.41)	5/96 (5.21)	5/82 (6.10)
	Syllable-final word-medial	3/43 (6.98)	9/205 (4.39)	0/39 (0)	—	—
	Syllable-final word-final	7/116 (6.03)	12/143 (8.39)	6/66 (9.09)	—	—
**U-TAP^g^**						
	Syllable-initial word-initial	14/349 (3.69)	1/117 (0.85)	1/10 (10)	0/70 (0)	5/78 (6.41)
	Syllable-initial word-medial	9/343 (2.62)	3/163 (1.84)	5/34 (14.71)	4/63 (6.35)	5/71 (7.04)
	Syllable-final word-medial	6/31 (19.35)	14/226 (6.19)	—	—	—
	Syllable-final word-final	6/81 (7.41)	10/181 (5.52)	9/75 (12)	—	—

^a^ASR: automatic speech recognition model.

^b^SLP: speech-language pathologist.

^c^The number of disagreements between the ASR and the SLPs/total number of cases flagged as correct articulations by the SLPs.

^d^APAC: Assessment of Phonology and Articulation for Children.

^e^Not applicable.

^f^Fricatives and affricates are not realized as final consonants in Korean.

^g^U-TAP: Urimal Test of Articulation and Phonology.

**Table 2:** Common ASR^a^ disagreements transcribed as correct articulations by the SLPs^b^.

Target words (meaning) [IPA^c^]			ASR disagreement type		Frequency, n/N^d^ (%)
**APAC^e^**					
	화장실 (toilet) [hwad 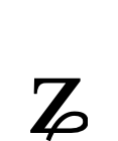 ɑŋ 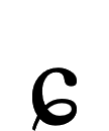 il]	Deletion of correctly sounded [l]		4/78 (5.13)	
	눈사람 (snowman) [nuns*ɑrɑm]	Deletion of correctly sounded [n]		3/78 (3.84)	
	눈사람 (snowman) [nuns*ɑrɑm]	Deletion of correctly sounded [m]		3/78 (3.84)	
	화장실 (toilet) [hwad 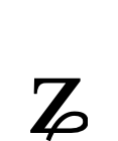 ɑŋ 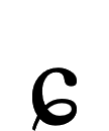 il]	Substitution of correctly sounded [d 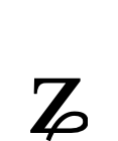 ] with [d]		3/78 (3.84)	
	컵 (cup) [k^h^ʌ 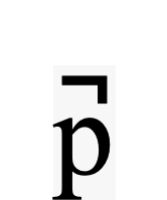 ]	Deletion of correctly sounded [ 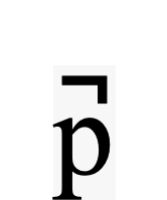 ]		3/78 (3.84)	
**U-TAP^f^**					
	짹짹 (tweet) [t 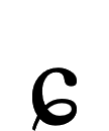 *ɛ 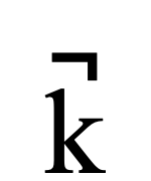 t 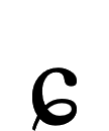 *ɛ 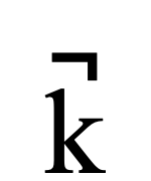 ]	Deletion of correctly sounded [ 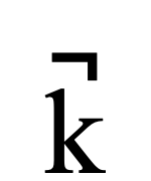 ] (medial sound)		4/92 (4.34)	
	그림 (drawing) [kɯrim]	Deletion of correctly sounded [k]		3/92 (3.26)	
	그림 (drawing) [kɯrim]	Deletion of correctly sounded [m]		3/92 (3.26)	
	귀 (ears) [kwi]	Substitution of correctly sounded [k] with [d 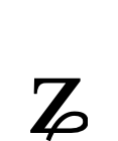 ]		3/92 (3.26)	
	바지 (pants) [pɑd 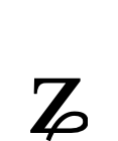 i]	Substitution of correctly sounded [d 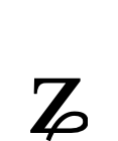 ] with [d]		3/92 (3.26)	

^a^ASR: automatic speech recognition model.

^b^SLP: speech-language pathologist.

^c^IPA: International Phonetic Alphabet.

^d^The number of specific ASR disagreements occurred/total number of ASR disagreements, specifically among cases flagged as correct articulations by the SLPs.

^e^APAC: Assessment of Phonology and Articulation for Children.

^f^ U-TAP: Urimal Test of Articulation and Phonology.

#### Disagreements in Misarticulations

The disagreements for phonemes transcribed as misarticulations by the SLPs averaged 7.87 (SD 3.66) and 7.57 (SD 4.85) occurrences per child, totaling 236 and 227 times among 30 children in the APAC and U-TAP, respectively. Table 3 presents the percentage of sounds that were judged by the SLPs as incorrectly produced but were identified as different by the ASR model. Table 4 lists the types of disagreements that occurred three or more times.

**Table 3:** The number of disagreements between the ASR^a^ and the SLPs^b^ for phonemes that were judged by the SLPs as incorrectly produced.

		Plosives, n/N^c^ (%)	Nasals, n/N (%)	Fluids, n/N (%)	Fricatives, n/N (%)	Affricatives, n/N (%)
**APAC^d^**						
	Syllable-initial word-initial	24/39 (61.54)	4/5 (80)	—^e^	20/67 (29.85)	11/36 (30.56)
	Syllable-initial word-medial	27/73 (36.99)	2/7 (28.57)	5/93 (5.38)	42/114 (36.84)	18/68 (26.47)
	Syllable-final word- medial	15/47 (31.91)	24/125 (19.20)	11/21 (52.38)	—	—
	Syllable-final word-final	11/34 (32.35)	12/37 (32.43)	10/24 (41.67)	—	—
**U-TAP^g^**						
	Syllable-initial word-initial	22/41 (51.22)	2/3 (66.67)	3/20 (15)	12/50 (24)	12/72 (16.67)
	Syllable-initial word-medial	22/47 (46.81)	7/17 (41.18)	19/146 (13.01)	22/87 (25.29)	13/49 (26.53)
	Syllable-final word-medial	13/29 (44.83)	34/134 (25.37)	—	—	—
	Syllable-final word-final	14/39 (35.90)	26/59 (44.07)	7/45 (15.56)	—	—

^a^ASR: automatic speech recognition model.

^b^SLP: speech-language pathologist.

^c^The number of disagreements between the ASR and the SLPs/total number of cases flagged as misarticulations by the SLPs.

^d^APAC: Assessment of Phonology and Articulation for Children.

^e^Not applicable.

^f^Fricatives and affricates are not realized as final consonants in Korean.

^g^U-TAP: Urimal Test of Articulation and Phonology.

**Table 4:** Common ASR^a^ disagreements transcribed as misarticulations by the SLPs^b^.

Target words (meaning) [IPA^c^]		SLPs’ judgment	ASR results	Frequency, n/N^d^ (%)
**APAC^e^**				
	딸기(strawberry) [t*ɑlgi]	[l] → Omitted	Recognizing misarticulation as correct articulation	6/236 (2.54)
	이빨 (teeths) [i̕p*ɑl]	[l] → Omitted	Recognizing misarticulation as correct articulation	5/236 (2.12)
	딸기 (strawberry) [t*ɑlgi]	[g] → [k*]	Recognizing misarticulation as correct articulation	5/236 (2.12)
	단추 (button) [tɑnt 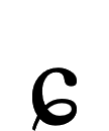 ^h^u]	[t 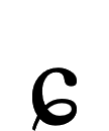 ^h^] → [t 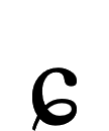 *]	Recognizing misarticulation as correct articulation	4/236 (1.69)
	눈사람(snowman) [nuns*aram]	[n] → Omitted	Recognizing misarticulation as correct articulation	4/236 (1.69)
**U-TAP^f^**				
	동물원 (zoo) [doŋmurwʌn]	[ŋ] → [m]	Recognizing misarticulation as correct articulation	6/227 (2.64)
	괴물(monster) [kwemul]	[l] → Omitted	Recognizing misarticulation as correct articulation	5/227 (2.2)
	눈썹 (eyebrow) [nuns*ʌ 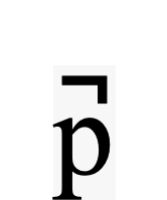 ]	[ 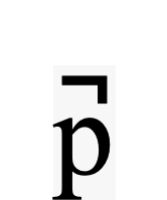 ] → Omitted	Recognizing misarticulation as correct articulation	4/227 (1.76)
	단추 (button) [tɑnt 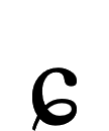 ^h^u]	[t 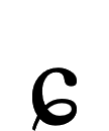 ^h^] → [t 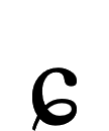 *]	Recognizing misarticulation as correct articulation	4/227 (1.76)
	짹짹 (tweet) [t 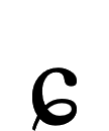 *ɛ 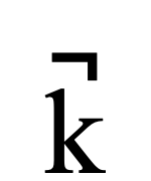 t 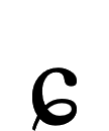 *ɛ 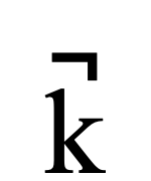 ]	[ 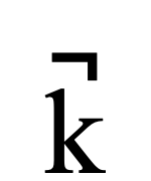 (final)] → Omitted	Recognizing misarticulation as correct articulation	4/227 (1.76)

^a^ASR: automatic speech recognition model.

^b^SLP: speech-language pathologist.

^c^IPA: International Phonetic Alphabet.

^d^The number of specific ASR disagreements occurred/total number of ASR disagreements, specifically among cases flagged as misarticulations by the SLPs.

^e^APAC: Assessment of Phonology and Articulation for Children.

^f^U-TAP: Urimal Test of Articulation and Phonology.

#### Target PCC

For the APAC, the mean target PCC was 74.76% (SD 15.21%) for SLPs and 76.71% (SD 15.20%) for the ASR model. The ICC for APAC demonstrated excellent reliability, with a value of 0.984 (95% CI 0.953-0.994). Similarly, for the U-TAP, the mean target PCC was 73.88% (SD 16.13%) for SLPs and 76.05% (SD 15.26%) for ASR. The ICC for U-TAP also indicated strong reliability, with a value of 0.978 (95% CI 0.941-0.990). The APAC uses 70 target consonants out of a total of 174 phonemes, whereas the U-TAP is based on 43 target consonants out of a total of 151 phonemes.

#### Severity

Table 5 displays the *z* scores for the target PCC in each test, as evaluated by the SLPs and ASR. The *z* scores between SLPs and ASR showed more pronounced differences with the APAC than with the U-TAP, with 8 individuals showing discrepancies in the APAC compared to only 2 in the U-TAP. The variations in *z* scores for each child are detailed in Multimedia Appendix 1.

**Table 5:** Comparison of children’s z scores assessed by the SLPs^a^ and the ASR^b^ model.

*z* score	APAC^c^		U-TAP^d^	
	SLPs^c^	ASR^d^	SLPs^c^	ASR^d^
Over +1	0	0	0	0
0 to 1	1	4	2	3
–1 to 0	3	3	0	0
–2 to –1	6	2	1	1
Under –2	17	18	24	23
Total^e^	27	27	27	27

^a^SLP: speech-language pathologist.

^b^ASR: automatic speech recognition model.

^c^APAC: Assessment of Phonology and Articulation for Children.

^d^U-TAP: Urimal Test of Articulation and Phonology.

^e^The APAC and U-TAP provide *z* scores specifically for children aged 2 to 6. Thus, 3 children who were aged 7 were excluded from the analysis of the *z* score comparisons.

## Discussion

### Overview

This study highlights the effectiveness of the end-to-end model in accurately capturing children’s articulations at the phoneme level. The PER between the SLPs and ASR model for the APAC and U-TAP assessments showed close correspondence (approximately 8%), with C-PER exhibiting similar ranges (ie, 10%-11%). Notably, the higher C-PER compared to the overall PER suggests fewer disagreements for vowels. Although the ASR model’s results did not perfectly align with the SLPs’ transcriptions, it is important to note that studies using standardized tests for SSDs typically report interrater reliability of 0.90 or higher, though it never reaches 100% [5,6,9,23]. Therefore, inherent variability in transcription, even among the SLPs, may have influenced the ASR evaluation process.

The occurrence of ASR disagreements in cases of correct articulation was relatively low, averaging approximately 2-3 instances per child. However, discrepancies in plosive sounds, particularly in the syllable-final word-medial position in the U-TAP, were notably higher compared to the APAC. This difference may stem from children imitating bird sounds when producing *짹짹* [t
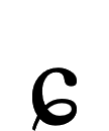
*ɛ
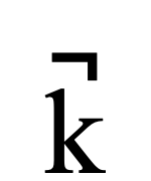
t
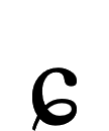
*ɛ
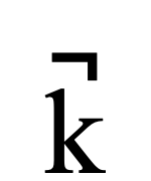
] (tweet, onomatopoeia) in the U-TAP, reducing transcription reliability, as seen in common ASR disagreements cases.

In contrast, disagreements between the SLPs and the ASR model for misarticulations were approximately three times higher than for correct articulations. This finding is consistent with Jing and Grigos [9], who noted that the reliability of evaluation diminishes further when assessing misarticulated phonemes rather than correctly articulated ones. Disagreements between the ASR and the SLPs were higher for nasals and plosives but lower for fricatives and affricates in both the APAC and U-TAP, particularly in the syllable-initial word-initial position. This discrepancy may be attributed to the limited dataset, as fricatives and affricates are developmentally acquired later, resulting in a greater opportunity for the model to learn more error data related to these sounds.

The results of this study suggest several implications for the selection of test words when using the ASR. First, test words with long and complex syllable structures, such as *옥수수* [o
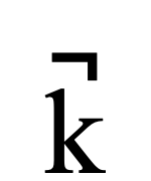
s*usu] (corn), *화장실* [hwɑd
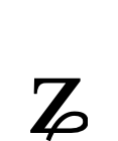
aŋ
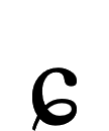
il] (bathroom), and *눈사람* [nuns*ɑram] (snowman) can hinder ASR systems in recognizing specific phonemes produced by children, particularly when the target phoneme is located in the word-medial position. Furthermore, accurate evaluation of the consonants ㅈ [t
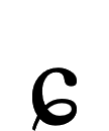
, d
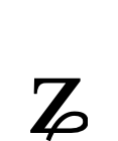
], ㄷ [t, d], and ㄱ [k, g] was challenging when followed by the vowel /i/, as seen in words like *바지* [pɑd
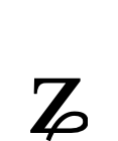
i] (pants) and *귀* [kwi] (ears), with ASR showing confusion between these phonemes. This difficulty is likely attributed to the proximity of their articulatory positions, which leads to similar auditory perceptual characteristics. In other words, assessing the accurate production of the preceding phoneme becomes particularly difficult when followed by the vowel /i/. In addition, the ASR demonstrated low performance in recognizing incorrect articulations of liquid sounds in syllable-final, word-medial positions. For example, it struggled to accurately detect the omission of the /l/ sound, as seen in words like *딸기* [t*ɑlgi] (strawberry) and *빨대* [p*ɑlt*ɛ] (straw).

Moreover, certain speech error patterns may not be accurately assessed using the ASR. First, it was difficult to distinguish whether plosive sounds at the end of a word were omitted or correctly produced (eg, *책* [t
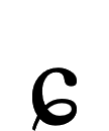
^h^ɛ
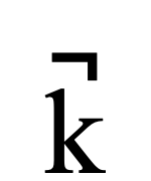
] (book)). Since syllable-final plosives are unreleased stops in Korean, the ASR may fail to accurately assess the speech of children who omit plosive consonants in the coda. Therefore, visual observation by SLPs should accompany the ASR evaluation to provide a more accurate assessment. Second, children exhibiting deaspiration errors may not be appropriately evaluated by the ASR, as misarticulation of aspirated consonants as tense consonants is difficult to detect (eg, *단추* [tɑnt
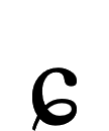
^h^u] vs [tɑnt
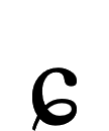
*u]; buttons). This highlights the limitations of ASR in capturing certain types of speech errors, underscoring the need for supplementary evaluation methods, such as human auditory judgment, to ensure accurate diagnosis.

In addition, it is essential to compare the PCC and *z* scores derived from the ASR recognition results, as these provide a more comprehensive analysis of its clinical usefulness. The reliability of the ASR model developed by Ahn et al [19], using both the APAC and U-TAP, was deemed excellent, as indicated by the target PCC, which showed a strong correlation with the SLPs’ evaluation results. However, this could be inflated with respect to diagnosing SSDs, as it is limited to results for only the targeted phoneme, rather than considering all phonemes in the test word.

When comparing the *z* scores for the target PCC, more pronounced differences were observed when using the APAC than when using the U-TAP (8 vs 2 individuals, respectively). This finding suggests that differences between the ASR model and SLPs may be more pronounced when using the APAC. These differences likely reflect variations in the normative data used for each test, with the inclusion of children with SSD being a key factor. Normative samples should include children with speech or language issues to avoid overidentification, placing normally developing children at the lower end of the distribution [24]. In this regard, the U-TAP included only “normal children,” while the APAC incorporated both “general children” and those suspected of having functional articulation and phonological disorders, though the exact number was unspecified. In the U-TAP, most children in this study were classified as severe cases with *z* scores below –2, which may explain the lack of significant change in *z* scores despite slight differences in ASR and SLP evaluations. This finding is also aligned with Yi and Kim [23], who compared *z* scores across different SSD assessment tools [23].

### Limitations and Suggestions

The limitations of this study include the use of a fine-tuned end-to-end model based on Korean phonemes, which may not be applicable for assessing speech errors in other languages or distortions not present in Korean.

Additionally, while machine learning relies on human transcriptions, it is essential to acknowledge that SLPs’ transcriptions are not always consistent. These discrepancies must be considered in the ASR’s training and evaluation, and future research should compare disagreement patterns among SLPs and between SLPs and the ASR.

In particular, this study conducted internal validation, meaning that both the model development and verification processes were carried out within the same setting. In addition, the small sample size makes it challenging to generalize the results. Therefore, external validation using other clinical settings or recording devices and including a larger number of participants is necessary.

For future studies, we suggest that incorporating connected speech tests may enhance the model’s applicability in real-world clinical settings. Additionally, integrating automated error pattern analysis programs into the ASR to evaluate and categorize children’s speech sound errors could streamline the evaluation process and save time for SLPs.

### Conclusions

This study has been the first to assess the efficacy of ASR at the phoneme level in discerning speech errors among children afflicted with SSDs in South Korea. The model exhibited a commendable reliability when compared to transcriptions provided by the SLPs. Thus, our findings clearly confirm the viability and potential of using such a model in the domain of speech-language pathology. Still, given that assessing children with SSDs is difficult solely through auditory perception, ASR cannot fully replace traditional evaluations.

## Appendix

Multimedia Appendix 1 Participant demographics and evaluation results.

Multimedia Appendix 2 Words list for the model training.

Multimedia Appendix 3 Target consonants and number of opportunities for production.

## Abbreviations

APAC: Assessment of Phonology and Articulation for Children
ASR: automatic speech recognition
C-PER: consonants phoneme error rate
HMM: hidden Markov model
ICC: intraclass correlation coefficient
PER: phoneme error rate
PCC: percentage of consonants correct
SLP: speech-language pathologist
SSD: speech sound disorder
U-TAP: Urimal Test of Articulation and Phonology

## Data Availability

The datasets generated during or analyzed during this study are not publicly available due to the fact that no prior consent was obtained from the participants to share their data but are available from the corresponding author on reasonable request.

## References

[1] Waring R, Knight R (2013). How should children with speech sound disorders be classified? A review and critical evaluation of current classification systems. Int J Lang Commun Disord.

[2] Kim SJ, Kim MJ, Ha S, Ha J (2015). A survey of speech sound disorders in clinical settings. Commun Sci Disord.

[3] Kim SJ, Ko YK, Seo EY, Oh GA (2017). Prevalence of speech sound disorders in 6-year-old children in Korea. Commun Sci Disord.

[4] Mcleod S, Baker E (2014). Speech-language pathologists' practices regarding assessment, analysis, target selection, intervention, and service delivery for children with speech sound disorders. Clin Linguist Phon.

[5] Kim M, Pae SY, Lee SE (2005). The development of the 'Test of Articulation for Children': concurrent validity. Commun Sci Disord.

[6] Kim Y, Shin M, Kim S, Ha J (2004). Urimal Test of Articulation and Phonology (U-TAP).

[7] Skahan SM, Watson M, Lof GL (2007). Speech-language pathologists' assessment practices for children with suspected speech sound disorders: results of a national survey. Am J Speech Lang Pathol.

[8] Keshet J (2018). Automatic speech recognition: a primer for speech-language pathology researchers. Int J Speech Lang Pathol.

[9] Jing L, Grigos MI (2022). Speech-language pathologists' ratings of speech accuracy in children with speech sound disorders. Am J Speech Lang Pathol.

[10] Cole RA (1973). Listening for mispronunciations: a measure of what we hear during speech. Percept Psychophys.

[11] Howard SJ, Heselwood BC (2002). Learning and teaching phonetic transcription for clinical purposes. Clin Linguist Phon.

[12] Kewley-Port D, Burkle TZ, Lee JH (2007). Contribution of consonant versus vowel information to sentence intelligibility for young normal-hearing and elderly hearing-impaired listeners. J Acoust Soc Am.

[13] Miller GA, Nicely PE (1955). An analysis of perceptual confusions among some English consonants. J Acoust Soc Am.

[14] Klein HB, Grigos MI, Byun TM, Davidson L (2012). The relationship between inexperienced listeners' perceptions and acoustic correlates of children's /r/ productions. Clin Linguist Phon.

[15] Dudy S, Bedrick S, Asgari M, Kain A (2018). Automatic analysis of pronunciations for children with speech sound disorders. Comput Speech Lang.

[16] Suanpirintr S, Thubthong N (2007). The effect of pauses in dysarthric speech recognition study on Thai cerebral palsy children.

[17] Mazenan MN, Swee TT, Soh SS (2014). Recognition test on highly newly robust Malay corpus based on statistical analysis for Malay articulation disorder. IEEE.

[18] Lee SH, Kim M, Seo HG, Oh BM, Lee G, Leigh JH (2019). Assessment of dysarthria using one-word speech recognition with hidden Markov models. J Korean Med Sci.

[19] Ahn T, Hong Y, Im Y, Kim DH, Kang D, Jeong JW, Kim JW, Kim MJ, Cho A, Nam H, Jang D (2024). Automatic speech recognition (ASR) for the diagnosis of pronunciation of speech sound disorders in Korean children. Clin Linguist Phon.

[20] McKechnie J, Ahmed B, Gutierrez-Osuna R, Monroe P, McCabe P, Ballard KJ (2018). Automated speech analysis tools for children's speech production: a systematic literature review. Int J Speech Lang Pathol.

[21] Baevski A, Zhou Y, Mohamed A, Auli M (2020). wav2vec 2.0: a framework for self-supervised learning of speech representations. Adv NeuralInf Process Syst.

[22] Landis JR, Koch GG (1977). The measurement of observer agreement for categorical data. Biometrics.

[23] Yi RD, Kim SJ (2019). Comparisons among single-word tests for Korean speech sounds and results of assessment for children with speech sound disorders. Commun Sci Disord.

[24] Dodd B, Holm A, Hua Z, Crosbie S (2003). Phonological development: a normative study of British English-speaking children. Clin Linguist Phon.

