# Supplementary material for: Usefulness of Automatic Speech Recognition Assessment of Children With Speech Sound Disorders: Validation Study
Source: J Med Internet Res. 2025 Jan 14;27:e60520. doi: 10.2196/60520 (PMC11775490; doi:10.2196/60520)
Supplement: Multimedia Appendix 2 [file jmir_v27i1e60520_app2.docx]

| Type | Words |
| --- | --- |
| APAC | 포도[pʰodo], 딸기[t*ɑlgi], 사탕[sɑtʰɑŋ], 햄버거[hɛmbʌgʌ], 옥수수[ok̚s*usu], 컵[kʰʌp̚], 빨대[p*ɑlt̕ɛ], 책[tɕʰɛk̚], 색종이[sɛk̚tɕ*oŋi], 머리[mʌri], 양말[yaŋmɑl], 단추[tɑntɕʰu], 모자[modʑɑ], 장갑[tɕɑŋgɑp̚], 빗[pit̚], 우산[usɑn], 침대[tɕʰimdɛ], 화장실[hwadʑɑŋɕil], 나무[nɑmu], 꽃[k*ot̚], 바퀴[pɑkʰɥi], 그네[kɯne], 시소[ɕiso], 눈사람[nuns*ɑrɑm], 토끼[tʰok*i], 이빨[ip*ɑl], 거북이[kʌbugi], 뱀[pɛm], 호랑이[horɑŋi], 고래[korɛ], 찢어[tɕ^*^idʑʌ], 싸워[s*ɑwʌ], 아파[ɑpʰɑ], 병원[pyʌŋwʌn], 안경[ɑngyʌŋ], 없어[ʌp̚s*ʌ], 올라가[ollɑgɑ] |
| U-TAP | 바지[pɑdʑi], 단추[tɑntɕʰu], 책상[tɕʰɛk̚s*ɑŋ], 가방[kɑbɑŋ], 사탕[sɑtʰɑŋ], 연필[yʌnpʰil], 자동차[tɕɑdoŋtɕʰɑ], 동물원[toŋmurwʌn], 엄마[ʌmmɑ], 뽀뽀[p*op*o], 호랑이[horɑŋi], 꼬리[k*ori], 코끼리[kʰok*iri], 땅콩[t*ɑŋkʰoŋ], 귀[kwi], 그네[kɯne], 토끼[tʰok*i], 풍선[pʰuŋsʌn], 로봇[robot̚], 그림[kɯrim], 못[mot̚], 눈썹[nuns*ʌp̚], 괴물[kwemul], 싸움[s*ɑum], 참새[tɕʰɑmsɛ], 세마리[semɑri], 짹짹[tɕ*ɛk̚tɕ*ɛk̚], 나무[nɑmu], 메뚜기[met*ugi], 전화[tɕʌnhwa] |
| Additional 12 words | 강아지[kɑŋɑdzi], 김밥[kimb*ɑp̚], 나비[nɑbi], 뚜껑[t*uk̕ʌŋ], 라면[rɑmyʌn], 버섯[pʌsʌt̚], 쓰레기[s*ɯregi], 양파[yaŋpʰɑ], 종이[tɕoŋi], 캥거루[kʰɛŋgʌru], 토마토[tʰomɑtʰo], 헬리콥터[heʎʎikʰop̚tʰʌ] |

APAC=Assessment of Phonology and Articulation for Children [5]; U-TAP=Urimal-Test of Articulation and Phonology [6]
