# Supplementary material for: Usefulness of Automatic Speech Recognition Assessment of Children With Speech Sound Disorders: Validation Study
Source: J Med Internet Res. 2025 Jan 14;27:e60520. doi: 10.2196/60520 (PMC11775490; doi:10.2196/60520)
Supplement: Multimedia Appendix 3 [file jmir_v27i1e60520_app3.docx]

|  | ㅂ  [p, b] | ㅃ  [p*] | ㅍ  [pʰ] | ㄷ  [t, d] | ㄸ  [t*] | ㅌ  [tʰ] | ㄱ  [k, g] | ㄲ  [k*] | ㅋ  [kʰ] | ㅅ  [s, ɕ] | ㅆ  [s*, ɕ*] | ㅎ  [h] | ㅈ  [tɕ, dʑ] | ㅉ  [tɕ*] | ㅊ  [tɕʰ] | ㅁ  [m] | ㄴ  [n] | ㄹ  [r, l] |
| --- | --- | --- | --- | --- | --- | --- | --- | --- | --- | --- | --- | --- | --- | --- | --- | --- | --- | --- |
| Word initial syllable initial |  |  |  |  |  |  |  |  |  |  |  |  |  |  |  |  |  |  |
| APAC | 4  [pit̚, pɛm] | 1  [p*ɑlt*ɛ]] | 1  [pʰodo] | 1  [tɑntɕʰu] | 1  [t*ɑlgi] | 1  [tʰok̕i] | 3  [kɯne, korɛ] | 1  [k*ot̚] | 1  [kʰʌp̚] | 3  [sɑtʰɑŋ, ɕiso] | 1  [s*ɑwʌ] | 3  [hɛmbʌgʌ, hwadzɑŋɕil] | 1  [tɕɑŋgɑp̚] | 1  [tɕ*idzʌ] | 2  [tɕʰɛk̚] | 2  [mʌri, modʑɑ] | 2  [nɑmu, nuns*ɑrɑm] | 0 |
| U-TAP | 1  [pɑdʑi] | 1  [p*o p*o] | 1  [pʰuŋsʌn] | 2  [tɑntɕʰu] | 1  [t*ɑŋkʰoŋ] | 1  [tʰok̕i] | 5  [kɯne] | 1  [k*ori] | 1  [kʰok̕iri] | 2  [sɑtʰɑŋ] | 1  [s*ɑum] | 1  [horɑŋi] | 2  [tɕɑdoŋtɕʰɑ] | 1  [tɕ*ɛk̚ tɕ*ɛk̚] | 2  [tɕʰɛk̚s̕ɑŋ] | 2  [mot̚] | 2  [nɑmu] | 1  [robot̚] |
| Word medial syllable initial |  |  |  |  |  |  |  |  |  |  |  |  |  |  |  |  |  |  |
| APAC | 2  [kʌbugi] | 1  [ip*ɑl] | 1  [ɑpʰɑ] | 2  [pʰodo, tɕʰimdɛ] | 1  [p*ɑl t*ɛ] | 1  [sɑtʰɑŋ | 6  [t*ɑlgi, tɕɑŋgɑp̚] | 1  [tʰok*i] | 1  [pa̠kʰɥi] | 4  [usɑn, ɕiso, hwadʑɑŋɕil] | 3  [ok̚s*usu, ʌp̚s*ʌ] | 0 | 3  [modʑɑ] | 1  [sɛk̚ tɕ*oŋi] | 1  [tɑntɕʰu] | 2  [nɑmu] | 1  [kɯne] | 5  [mʌri, horɑŋi,ollɑgɑ] |
| U-TAP | 2  [kɑbɑŋ] | 1  [p*o p*o] | 1  [jʌnpʰil] | 1  [tɕɑdoŋtɕʰɑ] | 1  [me t*ugi] | 1  [sɑtʰɑŋ | 1  [met*ugi] | 2  [kʰok*iri] | 1  [t̕ɑŋkʰoŋ] | 2  [pʰuŋsʌn] | 2  [nuns*ʌp̚] | 1  [tɕʌnhwa] | 1  [pɑdʑi] | 1  [tɕ*ɛk̚ tɕ*ɛk̚] | 2  [tɑntɕʰu] | 5  [nɑmu] | 1  [kɯne] | 6  [k*ori] |

|  | ㅂ  [p̚] | ㄷ  [t̚] | ㄱ  [k̚] | ㅁ  [m] | ㄴ  [n] | ㅇ  [ŋ] | ㄹ  [l] |
| --- | --- | --- | --- | --- | --- | --- | --- |
| word-medial syllable-final |  |  |  |  |  |  |  |
| APAC | 1  [ʌp̚s*ʌ] | 0 | 2  [ok̚ s*usu] | 2  [hɛmbʌgʌ, tɕʰimdɛ] | 3  [tɑntɕʰu, nuns*ɑrɑm] | 6  [jaŋmɑl, tɕɑŋgɑp, hwadʑɑŋɕil] | 3  [ollɑgɑ] |
| U-TAP | 0 | 0 | 2  [tɕʰɛk̚ s*ɑŋ] | 2  [ʌmmɑ] | 4  [tɕʌnhwa] | 5  [pʰuŋsʌn] ^a^ | 0 |
| word-final syllable-final |  |  |  |  |  |  |  |
| APAC | 2  [kʰʌp̚] | 2  [pit̚] | 1  [tɕ ʰɛk̚] | 2  [nuns*ɑrɑm] | 2  [usʰɑn] | 2  [sɑtʰɑŋ] | 3  [hwadʑɑŋɕil] |
| U-TAP | 1  [nuns*ʌp̚] | 2  [mot̚] | 1  [tɕ*ɛk̚tɕ*ɛk̚]^a^ | 2  [s*ɑum] | 2  [pʰuŋsʌn] ^a^ | 4  [kɑbɑŋ] | 2  [jʌnphil] |

[] Examples of test words

^a^Although it is included in the test words, it is not included in the target phoneme. Therefore, it is excluded when calculating PCC (Percent Consonants Correct).
